# EEG-Derived Transdiagnostic Subtypes Map Onto Distinct Brain-Behavior Dimensions

**DOI:** 10.1192/j.eurpsy.2026.10256

**Published:** 2026-07-31

**Authors:** J. Tabbal, A. Ebadi, G. Robert, A. Mheich, B. Rodríguez-Herreros, N. Chabane, A. Lefebvre, A. Iftimovici, S. Allouch, M. Hassan

**Affiliations:** 1 MINDIG; 2 U1228 Empenn UMR 6074 IRISA, Rennes, France; 3 Service des Troubles du Spectre de l’Autisme et apparentés, Département de psychiatrie, Lausanne University Hospital (CHUV), Lausanne, Switzerland; 4 Paris Saclay University, Neurospin, CEA Saclay, Service Avis et Expertise TND, Fondation Vallée; 5 Université Paris Cité, Institute of Psychiatry and Neuroscience of Paris (IPNP), INSERM U1266, Team “Pathophysiology of psychiatric disorders”, GDR 3557-Institut de Psychiatrie; 6 GHU Paris Psychiatrie et Neurosciences, Pôle hospitalo-universitaire d’évaluation, prévention, et innovation thérapeutique (PEPIT), Paris, France; 7 School of Science and Engineering, Reykjavik University, Reykjavik, Iceland

## Abstract

**Introduction:**

Psychiatric diagnoses traditionally rely on symptom-based classifications, which, despite providing a shared clinical and research framework, show weak alignment with underlying neurobiology. In response, data-driven, biologically informed approaches have emerged, yet most still depend on group-level comparisons that assume homogeneity and obscure individual variability. Normative modeling (NM) offers a promising alternative, quantifying individual deviations from population-derived normative distributions and enabling the detection of neurophysiological alterations at the single-subject level (Marquand et al. Biol Psychiatry 2016; 80:552-561).

**Objectives:**

This study aims to present a framework for clustering based on deviations from normative electrophysiological trajectories to identify transdiagnostic subtypes of neurodevelopmental and psychiatric disorders. The approach leverages a large, harmonized HD-EEG dataset to uncover biologically grounded brain–behavior profiles that transcend traditional diagnostic boundaries.

**Methods:**

We analyzed a multisite EEG dataset comprising 1,701 participants, including healthy controls, and individuals with attention-deficit/hyperactivity disorder, autism spectrum disorder, anxiety, learning disorders, and their comorbidities. Normative models were built for a comprehensive set of EEG features (n = 1957), and individualized deviation scores were computed. These multivariate deviation profiles were then clustered using similarity network fusion to identify subgroups with distinct electrophysiological patterns. The resulting clusters were subsequently characterized in terms of diagnostic composition and behavioral profiles.

**Results:**

We identified three neurophysiological subtypes that did not align neatly with traditional diagnostic categories (Fig. 1). Electrophysiologically, C2 (predominantly HC) showed deviation scores close to normative EEG trajectories, whereas C1 (internalizing symptoms) and C3 (externalizing diagnoses) exhibited more pronounced and often opposing deviations across key features. Behavioral comparisons revealed significant differences across behavioral domains (Fig. 2). C1 showed deficits in executive function and elevated emotional reactivity; C3 had higher hyperactivity-related scores; C2 showed the lowest scores across most domains, reflecting a broadly normative profile. Finally, EEG features significantly predicted cluster-specific behavioral traits (Fig. 3).

**Image 1:**
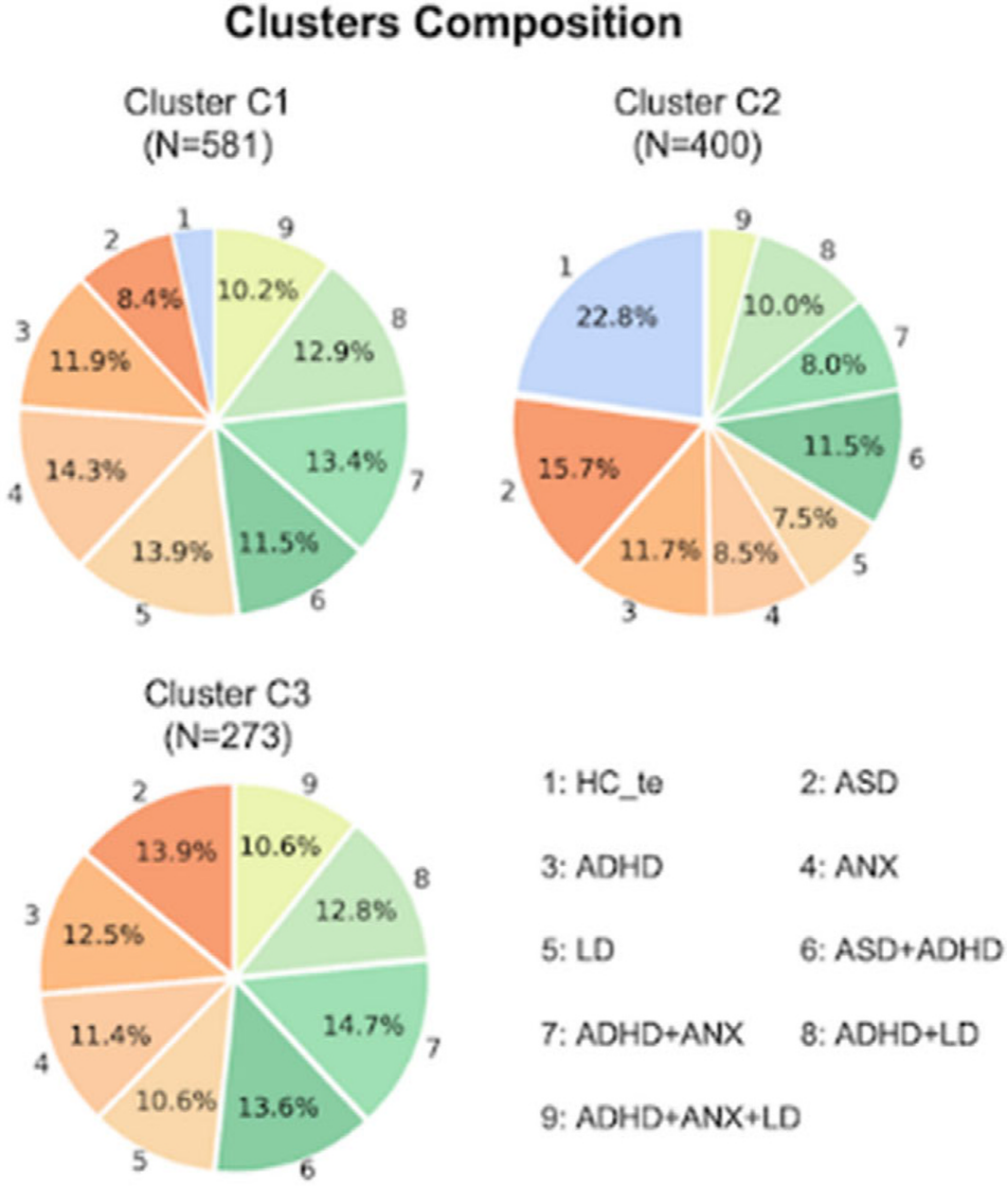
Image 1: Long description.

**Image 2:**
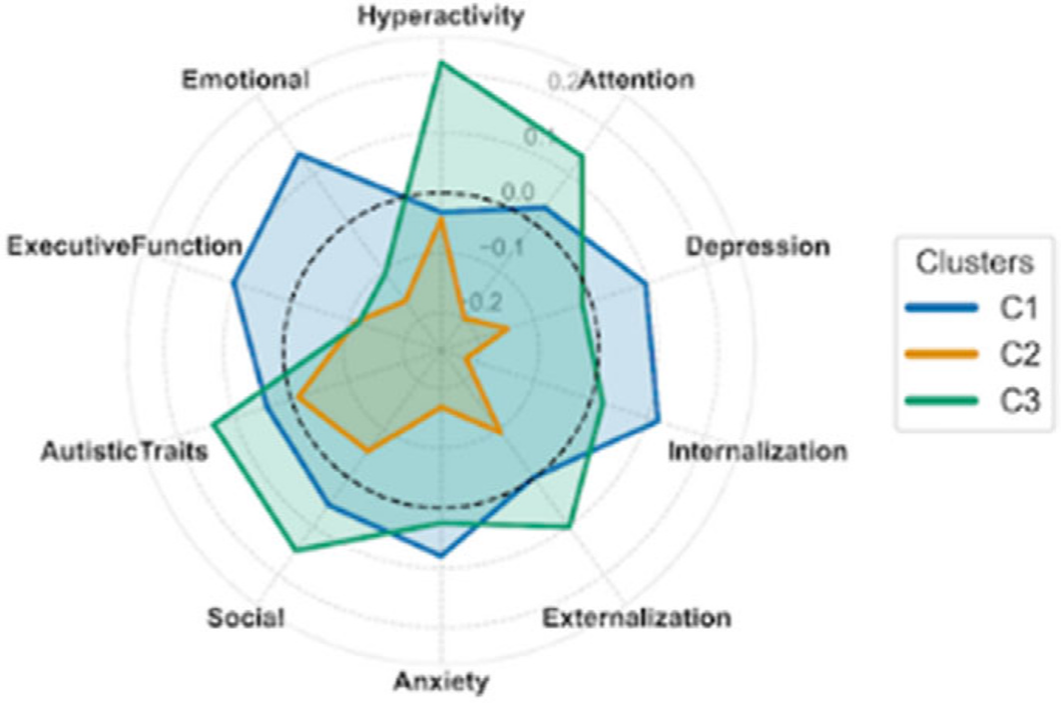
Image 2: Long description.

**Image 3:**
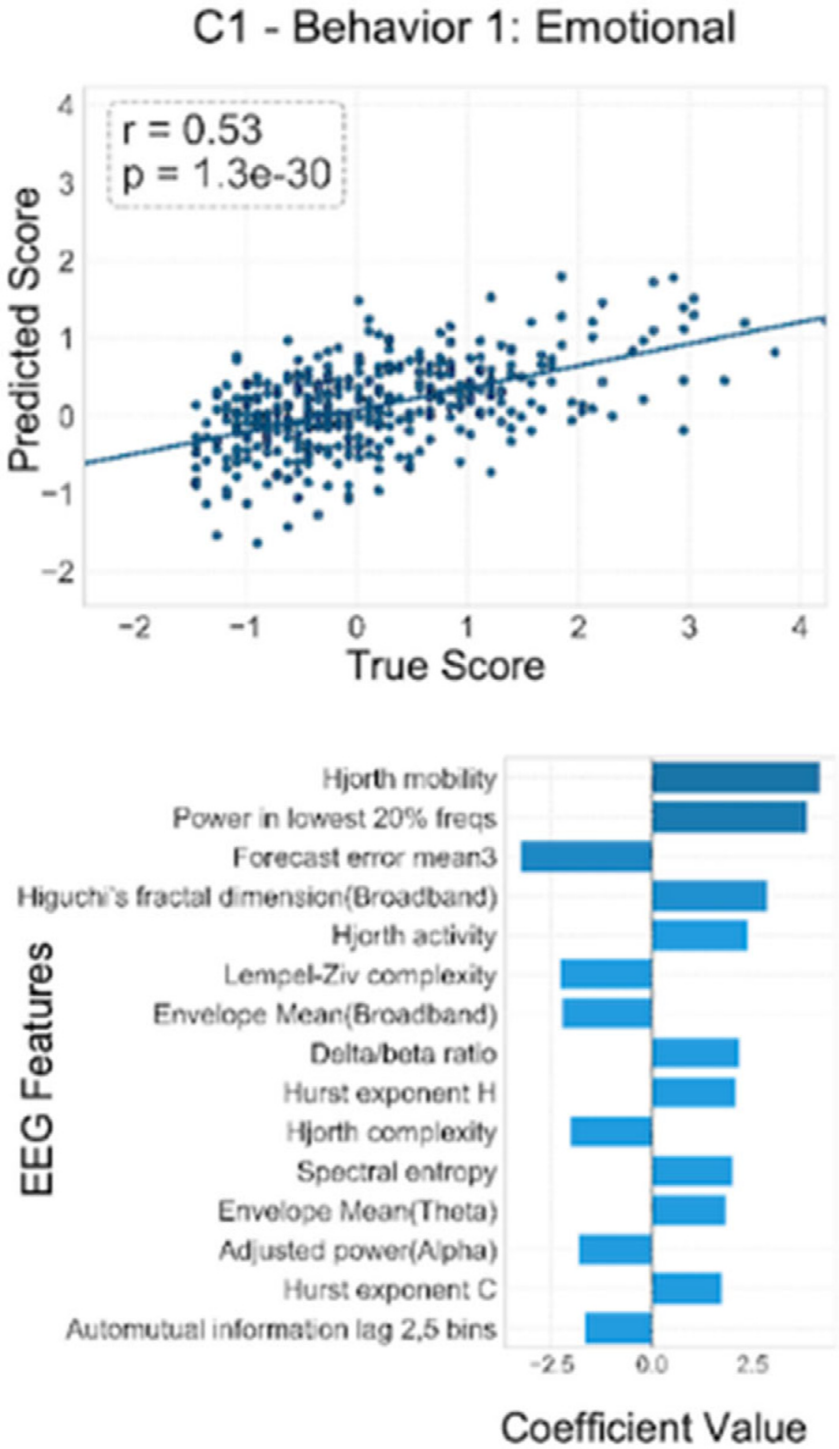
Image 3: Long description.

**Conclusions:**

Our EEG-based, data-driven stratification revealed transdiagnostic subtypes with distinct electrophysiological signatures, behavioral phenotypes, and brain-behavior associations. These biologically grounded profiles, which transcend traditional diagnostic boundaries, provide a principled framework to advance transdiagnostic research in psychiatry.

**Disclosure of Interest:**

None Declared

## Long descriptions

### Image 1: Long description

The figure consists of three pie charts and a legend.

Cluster C 1 (N equals 581) at the top left:

- 1: 2.8 percent

- 2: 8.4 percent

- 3: 11.9 percent

- 4: 14.3 percent

- 5: 13.9 percent

- 6: 11.5 percent

- 7: 13.4 percent

- 8: 12.9 percent

- 9: 10.2 percent

Cluster C 2 (N equals 400) at the top right:

- 1: 22.8 percent

- 2: 15.7 percent

- 3: 11.7 percent

- 4: 8.5 percent

- 5: 7.5 percent

- 6: 11.5 percent

- 7: 8.0 percent

- 8: 10.0 percent

- 9: 4.3 percent (estimated from slice size)

Cluster C 3 (N equals 273) at the bottom left:

- 2: 13.9 percent

- 3: 12.5 percent

- 4: 11.4 percent

- 5: 10.6 percent

- 6: 13.6 percent

- 7: 14.7 percent

- 8: 12.8 percent

- 9: 10.6 percent

Note: Category 1 is not visible in Cluster C 3.

Legend at the bottom right:

- 1: H C _ t e

- 2: A S D

- 3: A D H D

- 4: A N X

- 5: L D

- 6: A S D plus A D H D

- 7: A D H D plus A N X

- 8: A D H D plus L D

- 9: A D H D plus A N X plus L D

Navigate back to Image 1:.

### Image 2: Long description

A radar chart with ten axes arranged in a circle. Starting from the top and moving clockwise, the axes are Hyperactivity, Attention, Depression, Internalization, Externalization, Anxiety, Social, Autistic Traits, Executive Function, and Emotional. The radial scale ranges from negative 0.2 at the center to 0.2 at the outer edge, with a dashed black circle representing the 0.0 baseline.

* Cluster C 1 (blue line) shows high values in Executive Function and Emotional categories, moderate values in Depression and Internalization, and low values in Hyperactivity and Attention.

* Cluster C 2 (orange line) is the smallest, remaining mostly within the negative 0.1 to 0.0 range across all categories, with its highest relative point in Hyperactivity.

* Cluster C 3 (green line) shows a significant peak in Hyperactivity and Attention, with high values in Autistic Traits and Social categories, but lower values in Depression and Internalization.

Navigate back to Image 2:.

### Image 3: Long description

The top panel is a scatter plot titled C 1 - Behavior 1 Emotional. The x-axis is True Score and the y-axis is Predicted Score, both ranging from -2 to 4. A dense cluster of blue data points shows a positive linear correlation. A regression line is drawn through the data. A text box in the top-left corner indicates r = 0.53 and p = 1.3 e - 30.

The bottom panel is a horizontal bar chart titled E E G Features on the y-axis and Coefficient Value on the x-axis. The x-axis ranges from -2.5 to 2.5.

Positive coefficients from highest to lowest:

* Hjorth mobility: approximately 4.0

* Power in lowest 20% freqs: approximately 3.8

* Higuchi's fractal dimension Broadband: approximately 2.8

* Hjorth activity: approximately 2.2

* Delta/beta ratio: approximately 2.1

* Hurst exponent H: approximately 2.0

* Spectral entropy: approximately 1.9

* Envelope Mean Theta: approximately 1.8

* Hurst exponent C: approximately 1.7

Negative coefficients from most negative to least negative:

* Forecast error mean 3: approximately -2.5

* Hjorth complexity: approximately -1.9

* Adjusted power Alpha: approximately -1.8

* Lempel-Ziv complexity: approximately -1.5

* Envelope Mean Broadband: approximately -1.4

* Automutual information lag 2,5 bins: approximately -1.3

Navigate back to Image 3:.

